# Precision Genome Engineering in *Streptococcus suis* Based on a Broad-Host-Range Vector and CRISPR-Cas9 Technology

**DOI:** 10.1021/acssynbio.3c00110

**Published:** 2023-08-21

**Authors:** Alex Gussak, Maria Laura Ferrando, Mels Schrama, Peter van Baarlen, Jerry Mark Wells

**Affiliations:** Host-Microbe Interactomics, Animal Sciences, Wageningen University, 6708 WD Wageningen, The Netherlands

**Keywords:** CRISPR-Cas9, genetic engineering, Streptococcus suis, zoonotic, pathogen, persister cells

## Abstract

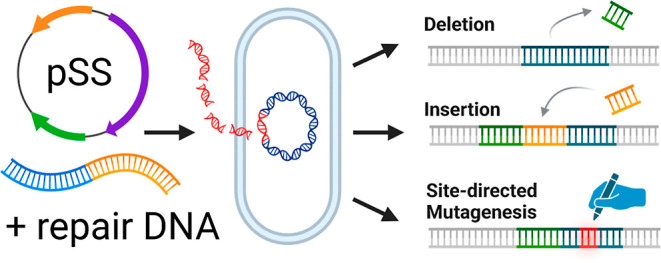

*Streptococcussuis* is an important zoonotic pathogen that causes severe invasive disease in pigs and humans. Current methods for genome engineering of *S. suis* rely on the insertion of antibiotic resistance markers, which is time-consuming and labor-intensive and does not allow the precise introduction of small genomic mutations. Here we developed a system for CRISPR-based genome editing in *S. suis*, utilizing linear DNA fragments for homologous recombination (HR) and a plasmid-based negative selection system for bacteria not edited by HR. To enable the use of this system in other bacteria, we engineered a broad-host-range replicon in the CRISPR plasmid. We demonstrated the utility of this system to rapidly introduce multiple gene deletions in successive rounds of genome editing and to make precise nucleotide changes in essential genes. Furthermore, we characterized a mechanism by which *S. suis* can escape killing by a targeted Cas9-sgRNA complex in the absence of HR. A characteristic of this new mechanism is the presence of very slow-growing colonies in a persister-like state that may allow for DNA repair or the introduction of mutations, alleviating Cas9 pressure. This does not impact the utility of CRISPR-based genome editing because the escape colonies are easily distinguished from genetically edited clones due to their small colony size. Our CRISPR-based editing system is a valuable addition to the genetic toolbox for engineering of *S. suis*, as it accelerates the process of mutant construction and simplifies the removal of antibiotic markers between successive rounds of genome editing.

## Introduction

*Streptococcus suis* is one of the major porcine bacterial pathogens and a common colonizer of the porcine upper respiratory tract, the gastrointestinal tract, and the genital tract.^1^ Although carriage of *S. suis* is endemic in farmed pigs, it mainly causes an invasive disease in young piglets around weaning. *S. suis* infections may lead to meningitis, arthritis, and septicaemia.^2–4^*S. suis* is also a zoonotic pathogen causing sepsis, meningitis (with deafness as a consequence), and endocarditis in humans.^5^ Risk factors include close contact with infected animals or carcasses and the consumption of raw pork products.^6–8^ Human-to-human transmission of *S. suis* has not been reported. Within the species *S. suis*, different serotypes and pathotypes have been identified, ranging from highly virulent to nonvirulent strains.^4^ Serotype 2 has frequently been reported to contain virulent strains, and most zoonotic strains belong to this serotype.^9^

To investigate *S. suis* virulence mechanisms and enhance our understanding of its pathobiology via mutant analysis, it is crucial that its genome can be efficiently edited. A recent advance in the genetic engineering of *S. suis* was the discovery of a peptide pheromone-inducible mechanism of extracellular DNA uptake (competence) and DNA integration into the genome.^10,11^ This method enables the high-efficiency genetic transformation of many *S. suis* strains. However, the obligatory use of antibiotic selection markers limits the number of possible genomic edits, and the insertion of a selection marker gene and promoter may cause polar effects on neighboring genes, requiring complementation approaches to confirm that the genetic deletion is responsible for an observed phenotype. While combined selection-counterselection methods have been used for marker-less mutant generation, these systems usually require time-consuming procedures involving multiple steps to remove the selection marker.^12^ Moreover, certain mutations, such as single nucleotide substitutions, cannot be constructed using these methods.

A recent development in the field of genome engineering was the advent of CRISPR-Cas9 technology, which allows targeted genome modification with unprecedented accuracy. CRISPR-Cas is a family of adaptive immune systems that is present in many prokaryotes.^13^ Naturally, this system acts as a defense mechanism against specific invading DNA elements such as plasmids, mediated via small RNAs—encoded in the CRISPR locus—that match corresponding sequences in the invading DNA.^14^ In type II CRISPR systems, the Cas9 enzyme cleaves invading DNA in a sequence-specific manner.^14,15^ Cas9 is guided to its target DNA sequence by a dual RNA complex consisting of a CRISPR RNA (crRNA) that contains a target-specific spacer sequence and a trans-activating crRNA (tracrRNA) that plays a role in the maturation of crRNA. A protospacer adjacent motif (PAM) of 2–6 nt must immediately follow the target sequence to enable binding and cleavage by Cas9. Thus, the PAM sequence is an essential targeting motif distinguishing “nonself” (a sequence in the invading DNA) from “self” (the same sequence in the genomic CRISPR spacer lacking a PAM). To exploit this system for genome editing, the crRNA-tracrRNA complexes can be engineered into a single fusion guide RNA via a flexible linker, resulting in a chimeric single guide RNA (sgRNA). Since most prokaryotes lack a nonhomologous end-joining mechanism for DNA repair, they are unable to repair their chromosomal DNA after cleavage by Cas9, leading to cell death.^16,17^ This can be utilized for genome editing by providing a repair template (RT), which is a homologous DNA fragment containing the desired modifications but lacking the sequence that is targeted by Cas9. By incorporation of the modified DNA fragment into the genome via homologous recombination (HR), the bacteria can evade Cas9-mediated DNA cleavage and subsequent cell death.

Here, we describe the construction of a broad-host-range shuttle vector (pSStarget) expressing *Streptococcus pyogenes* Cas9 and demonstrate how this vector can be used for efficient genome editing in *S. suis*. The pSStarget vector facilitates highly efficient sgRNA cloning via replacement of a negative selection marker, and is easily lost from *S. suis* cells when cultured in the absence of antibiotic pressure. We show that pSStarget can be used for multiple successive rounds of high-precision Cas9-mediated genome editing in *S. suis* without genomic integration of selective markers. Moreover, we demonstrate the application of our pSStarget-based CRISPR-Cas system to construct a mutant with a single amino acid substitution in the essential *eno* gene. Finally, we describe small colony variants (SCVs) that are seemingly resistant to Cas9-mediated genome editing in the absence of a RT. We propose a potential mechanism for the recovery of SCVs based on RNaseq and subsequent growth studies.

## Results

### Construction of pSStarget

We used the previously published pLABtargetc vector, which was originally developed for CRISPR-targeting in *Lactococcus lactis*,^18^ as a starting point for the construction of pSStarget. The broad-host-range pIL253 origin of replication (ori) allows its use in a wide variety of Gram-positive organisms, including lactobacilli, bacilli, and *Streptococcus* species.^19–22^ pLABtargetc contains a chloramphenicol acetyltransferase (*cat*) gene conferring chloramphenicol resistance, wild-type (wt) CRISPR-associated endonuclease (*cas9*) from *S. pyogenes* under the control of its native promoter, and an sgRNA handle (the portion that binds Cas9) with BsaI restriction sites that can be used for cloning of a specific spacer sequence. Expression of sgRNA is driven by the lactococcal *eps* promoter.

**Figure 1:**
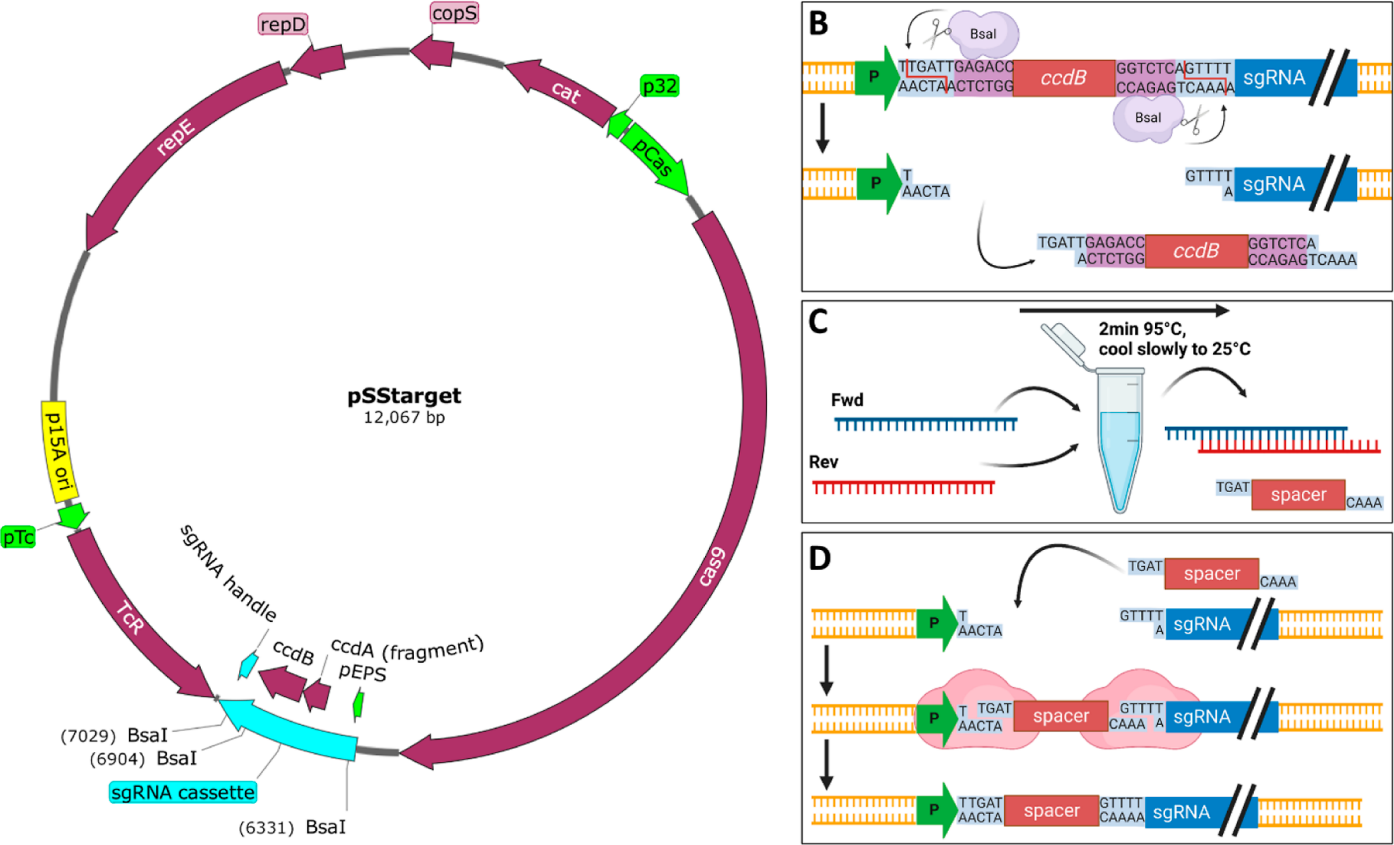
Overview of pSStarget and the sgRNA cloning strategy (A) plasmid map of pSStarget showing the main plasmid features. Protein coding sequences are shown in purple, promoters in green, and the p15A ori in yellow. The sgRNA expression cassette, which is divided into subparts, is colored light blue. The sgRNA expression cassette contains three *BsaI* sites but for clarity the internal one was omitted from panels (B–D) Schematic representation of the sgRNA cloning strategy. The *BsaI* restriction and ligation steps are depicted separately but can be combined in a single reaction tube because the correctly assembled constructs lack the *BsaI* sites. (B) Close-up of the sgRNA expression cassette. The *BsaI* restriction enzyme recognizes its binding site (purple) and cleaves the DNA, thereby removing the *ccdB* toxin gene together with the *BsaI* sites and generating 4 bp overhangs at the end of the linearized plasmid. (C) Annealing of two partially complementary oligonucleotides generates a dsDNA fragment encoding the spacer sequence with 4 bp overhangs on each side that are complementary to those on the linearized plasmid. (D) The spacer fragment is ligated into the sgRNA expression cassette, making use of the matching overhangs.

To generate a shuttle vector for cloning in *Escherichia coli*, we cloned the Gram-negative p15A ori and a tetracycline resistance marker (*tetA*) into pLABtargetc, resulting in the plasmid pSStarget-NT. The p15A ori has a low copy number in *E. coli*, minimizing the toxic effects of Cas9 expression during cloning. To facilitate efficient cloning of the spacer sequence, we inserted the negative selection marker *ccdB* (encoding a toxin protein which selectively inhibits DNA gyrase in the *E. coli*cloning host) between two BsaI restriction sites to generate the plasmid pSStarget (Figure 1A). Target-specific spacer sequences were generated by annealing partially complementary oligonucleotides, resulting in 4 nt overhangs that anneal to the overhangs generated by BsaI digestion of pSStarget (Figure 1C). Insertion of the spacer sequence eliminates the BsaI sites and removes the *ccdB* gene (Figure 1B,D). The CcdB-resistant *E. coli*strain ccdB Survival 2 was used for propagation of the pSStarget plasmid.

### Cas9-Mediated Genome Editing and “CRISPR Escape” Mutants

**Figure 2:**
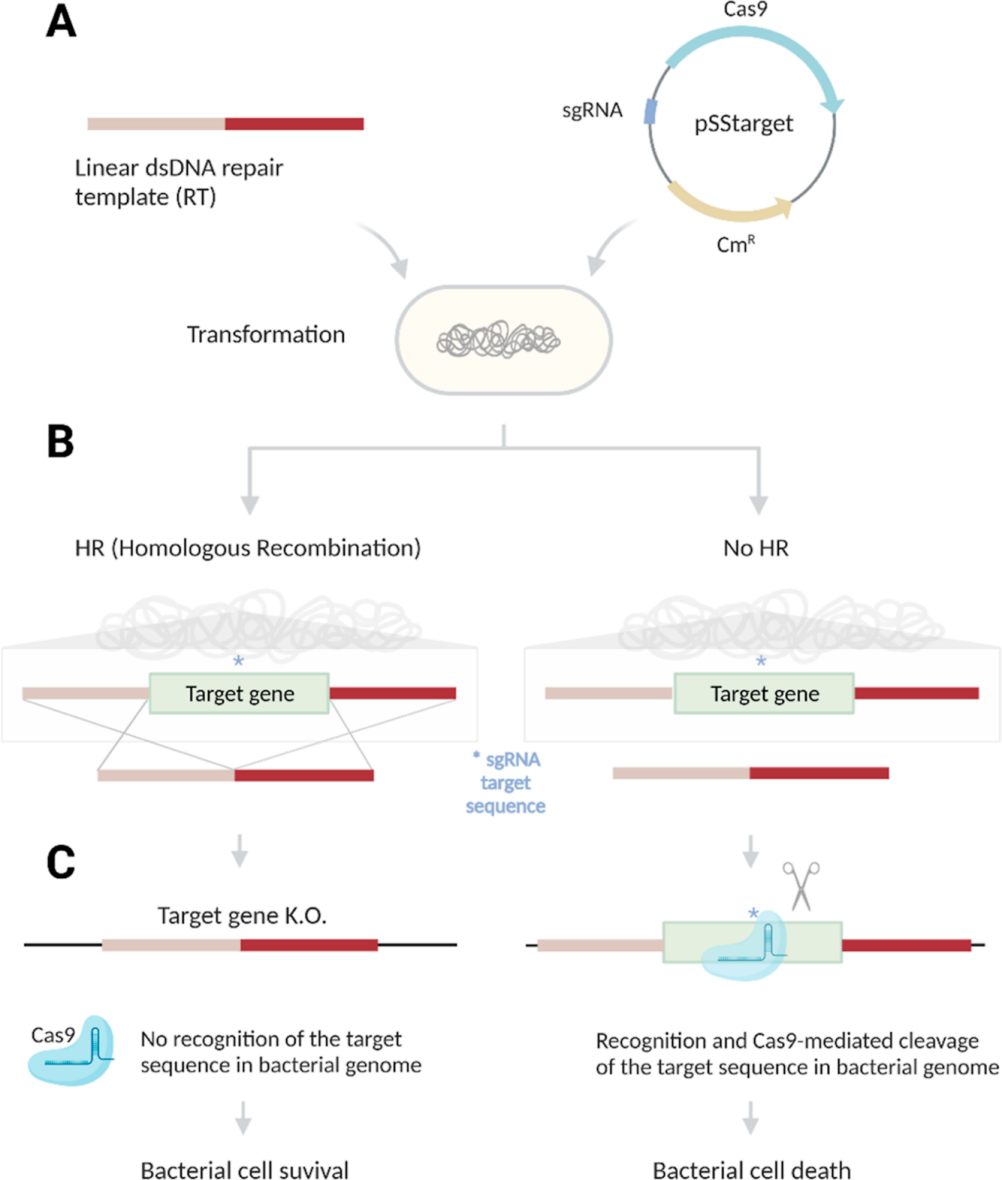
Schematic overview of the principle behind Cas9-mediated genome editing in *S. suis*. (A) *S. suis* is cotransformed with plasmid pSStarget and a linear repair template (RT) (B) homologous recombination of the RT with the chromosome occurs in a subset of the transformed cells. Meanwhile, Cas9 and sgRNA are expressed and assembled to form a complex. (C) Mature Cas9/sgRNA complex scans the genome for target sites. Edited cells that incorporated the RT by recombination lack the target site, making them immune to Cas9-mediated DNA cleavage. In the absence of HR, the target site is cleaved by Cas9, leading to cell death.

The use of the CRISPR-Cas9 system for genome engineering relies on HR carried out by *S. suis* using a linear RT, followed by Cas9-mediated counter-selection (Figure 2). RTs contained the modified target region (e.g., deletion or single nucleotide changes) flanked by approximately 1000 bp of homologous DNA on each side to allow for efficient HR in the corresponding *S. suis* genome. Transformation of bacteria with the RT and a target-specific pSStarget vector will lead to HR in a subset of cells. This results in the genomic integration of the RT and the simultaneous loss of the WT sequence containing the target site for the sgRNA. In cells that do not incorporate the RT into the genome, the Cas9/sgRNA complex cleaves DNA sequences matching the target-specific sgRNA, leading to double-stranded breaks in the genome. Therefore, Cas9 can be utilized as an efficient negative counterselection tool, as double-stranded breaks in genomic DNA are lethal to most bacteria.

To demonstrate the use of this system for genome engineering in *S. suis*, we chose three previously characterized virulence factors with known deletion phenotypes: (1) the *cpsE* and *cpsF* genes that are part of the capsular polysaccharide locus; (2) the gene encoding the hemolytic toxin Suilysin (*sly*); and (3) the *lgt* gene, which encodes the Lgt transferase involved in anchoring lipoproteins to the cell membrane.

**Figure 3:**
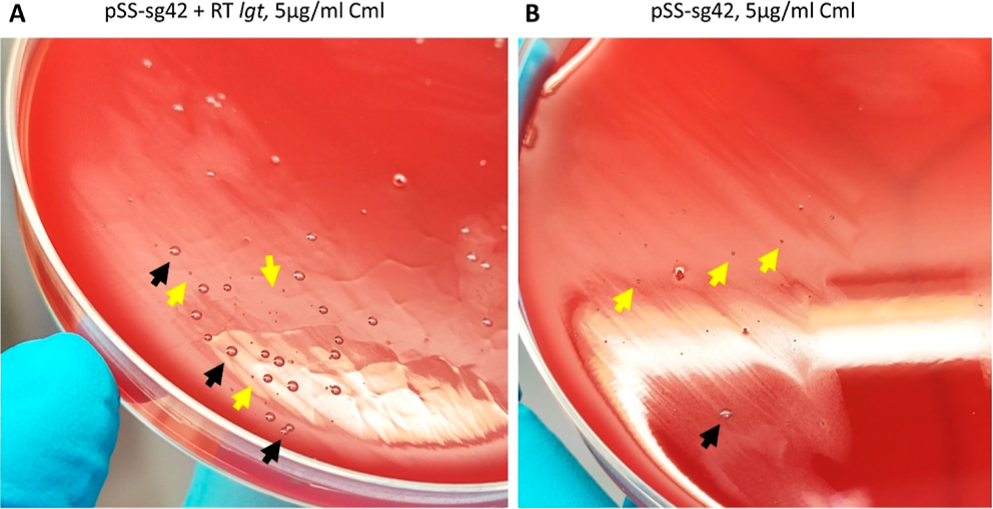
Representative pictures of colonies observed after plating the transformation mix on Columbia Blood Agar (CBA) supplemented with 5% sheep blood and 5 μg/mL chloramphenicol (Cml). Normal-sized colonies are indicated with black arrows, and SCVs are indicated using yellow arrows. (A) *S. suis* P1/7 transformed with pSS-sg42 and the corresponding repair template (1.2 μg each) forms many SCVs besides the normal-sized colonies. (B) *S. suis* P1/7 transformed only with pSS-sg42 (1.2 μg) was used as a “background control” to assess the amount of SCVs formed in the absence of a corresponding repair template. Mainly, SCVs are formed in the absence of an RT, and only a single normal-sized colony was observed.

To our surprise, we observed two different colony morphologies upon transformation of *S. suis* P1/7 with pSStarget plasmids containing a target sgRNA and corresponding RT, but not with a nontargeting plasmid lacking a sgRNA spacer (pSStarget-NT). Transformation of *S. suis* with pSStarget-NT yielded approximately 3.2 × 10^4^ normal-sized colonies. Co-transformation of *S. suis* with pSStarget containing target-specific sgRNAs and their corresponding RTs yielded a mixture of normal-sized colonies and small colonies that were barely visible to the naked eye (Figure 3A). When *S. suis* was transformed with only the pSStarget-sgRNA plasmids, predominantly small colony variants (SCVs) were recovered (Figure 3B). The number of small colonies recovered was highly dependent on the sgRNA sequence, with sg6 yielding many more small colonies than sg4 and sg42, giving rise to an intermediate amount of small colonies (data not shown). Moreover, small colony counts were lower when a corresponding RT was cotransformed than when only pSStarget was transformed.

More than 80% of the normal-sized colonies contained the desired deletion, confirmed by colony PCR. It was not always possible to generate an amplicon from small colonies, presumably due to the low number of bacteria. When a PCR amplicon was obtained from SCVs transformed with pSStarget-sg4, the amplicon size was approximately 3.5 kb, corresponding to the length expected for the WT *sly* sequence. As no mutations were identified in the target site amplicons, we hypothesized the SCVs were able to resist Cas9-mediated killing by an unknown mechanism. Although large numbers of the small “CRISPR-tolerant” SCVs can be present after transformation, it does not pose a problem for mutant selection, as SCVs are easily distinguished from the large colonies of bacteria with successfully edited genomes.

### Rapid Loss of pSStarget under Nonselective Growth Conditions Enables Multiple Rounds of Genome Editing

We then sought to investigate the possibility of generating double-knockout mutants by conducting successive rounds of genome editing. To allow the transformation of a new pSStarget plasmid targeting a different gene, the obtained mutants first had to lose the original pSStarget plasmid to restore their sensitivity to chloramphenicol. To achieve this, we grew the obtained mutants overnight in a large volume (30 mL) of THY in the absence of antibiotics. The next day an aliquot of the culture was spread on selective agar plates to verify the loss of the plasmid. In most cases (>90%), all cells had lost the plasmid after one night of culture. In the remaining cases, the plasmid was lost after a second night of culture. The Δ*cps* mutant was used as a parental strain for the construction of two double-knockout strains, resulting in strains Δ*cps* Δ*sly* and Δ*cps* Δ*lgt*.

**Figure 4:**
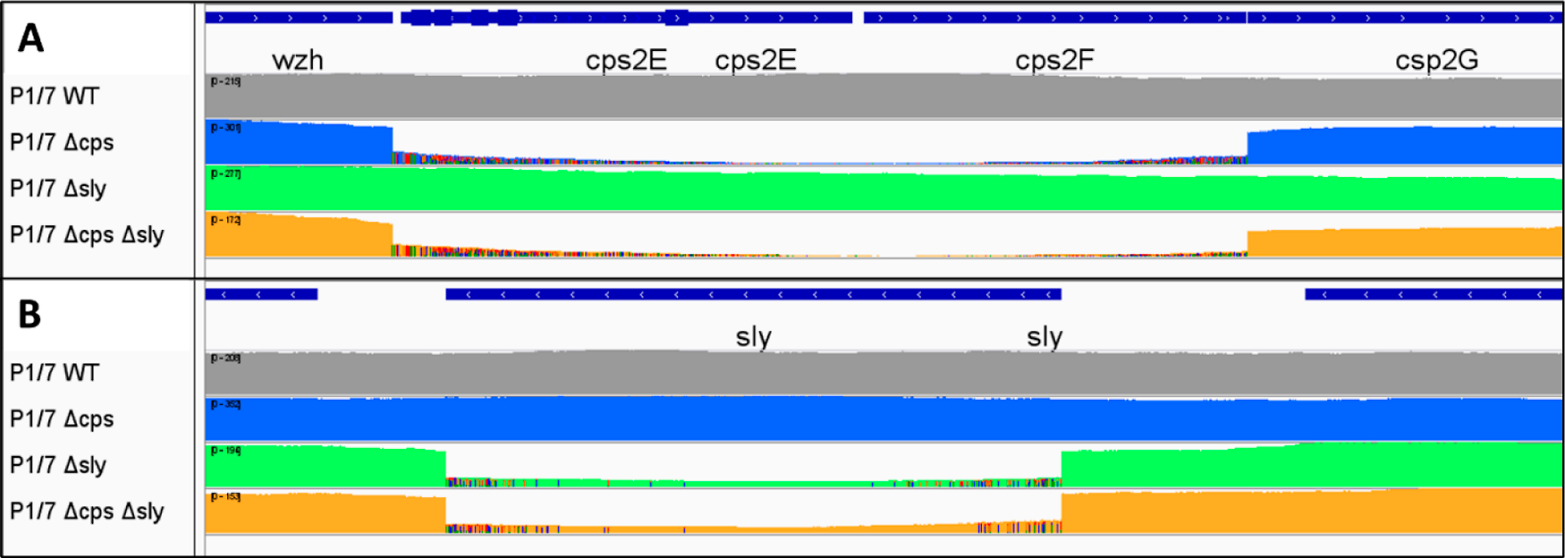
Coverage graph showing the alignment of ONT long-read sequencing data generated for strain P1/7 and the mutant strains Δ*cps*, Δ*sly*, and Δ*cps* Δ*sly* to the *cps* (A) and *sly* (B) loci of the reference sequence for strain P1/7 (AM946016.1). The read coverage drops substantially in the regions targeted for deletion, confirming successful genome editing.

To demonstrate the absence of off-target effects during CRISPR-Cas9-mediated genome engineering, we sequenced the whole genome of the CRISPR-edited strains Δ*cps*, Δ*sly*, and Δ*cps* Δ*sly*, as well as the genome of the parental strain P1/7. We identified a deletion of 5 bp in an intergenic region (660,128–660,132, GCAGA) and two SNPs in our laboratory stock of P1/7 relative to the published P1/7 reference sequence (AM946016.1). One mutation (1151748A > T) is located in an intergenic region, and the second (1406781G > T) results in an amino acid change (G47 V) in gene SSU1381. When comparing the mutant strains with our new P1/7 sequence, the exact deletion of the target regions was confirmed, and no additional mutations or structural rearrangements were found, demonstrating the high fidelity and accuracy of our CRISPR-Cas9 genome editing procedure. (Figure 4)

### Phenotyping of Selected Mutants

**Figure 5:**
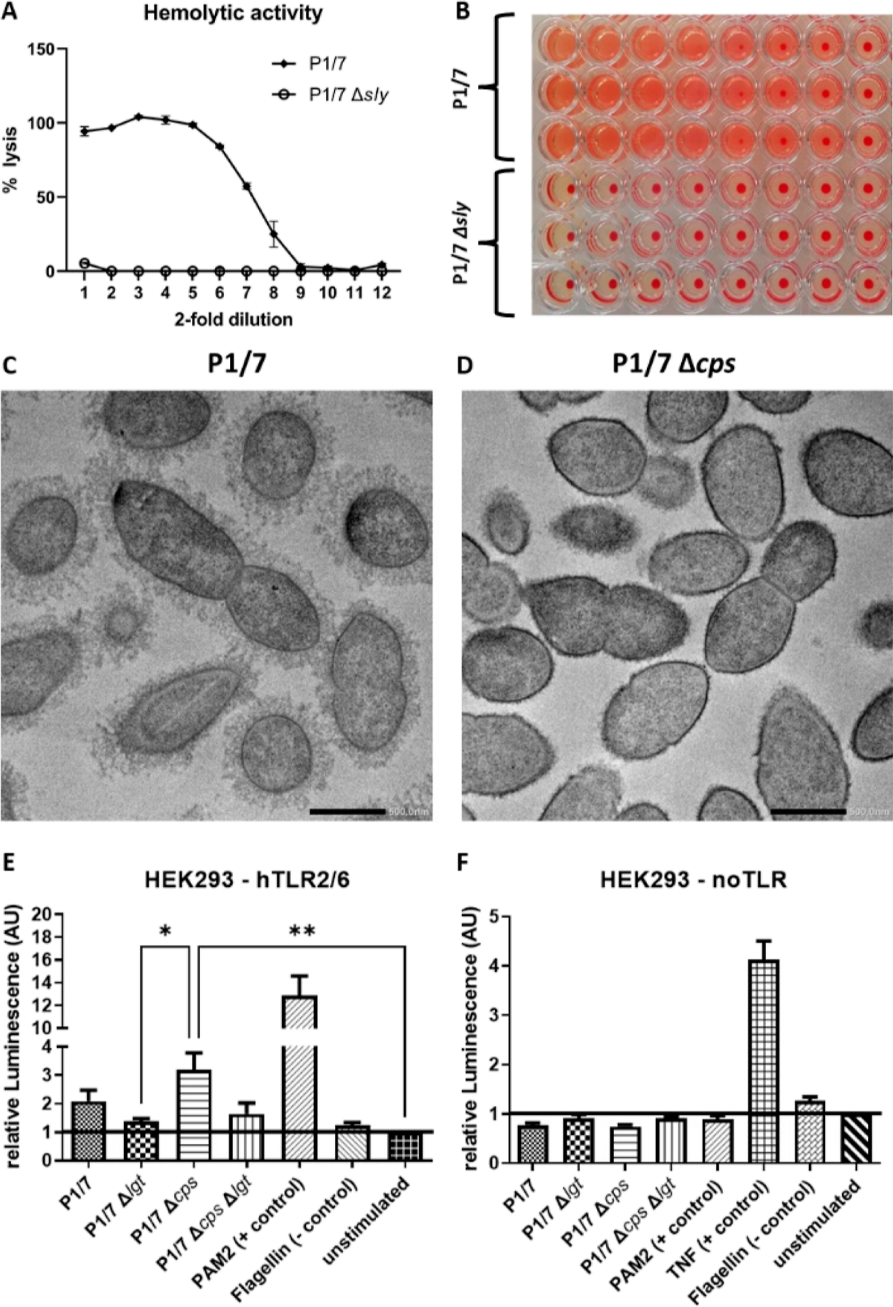
Phenotypic characterization of mutant strains. (A) Graph showing hemolytic activity of *S. suis* P1/7 and P1/7 Δ*sly*. All values are the means of three experiments. Values are expressed as percent lysis relative to the complete lysis control (1% Triton X-100). (B) Image of a representative hemolysis assay plate. (C) Representative electron microscopy image of the WT P1/7 at 12,000× magnification. The polysaccharide capsule is clearly visible surrounding the bacterial cells. (D) Representative electron microscopy image of strain P1/7 Δ*cps* at 12,000× magnification. No polysaccharide capsule is present on the surface of the cells, confirming the successful deletion of the *cpsEF* genes. (E) Quantification of NF-κB signaling resulting from hTLR2/6 activation by *S. suis* P1/7 and the Δ*cps*, Δ*lgt*, *and* Δ*cps* Δ*lgt* mutant strains. Deletion of the *lgt* gene resulted in decreased hTLR2/6 activation, whereas deletion of the *cpsEF* gene resulted in increased hTLR2/6 activation. Results are shown as relative luminescence values, with values of unstimulated hTLR2/6 cells set to 1. (F) Quantification of NF-κB signaling by *S. suis* P1/7 and the Δ*cps*, Δ*lgt*, and Δ*cps* Δ*lgt* mutant strains in control cells lacking TLRs. No differences between the tested strains were observed in this cell line, confirming the absence of TLR-independent NF-κB signaling. Results are shown as relative luminescence values, with values of unstimulated cells set to 1.

We conducted multiple assays to confirm that the constructed mutants had the expected phenotypes (Figure 5). For the Δ*sly* mutant, we assayed hemolytic activity after growth in pullulan, which was previously shown to induce *sly* expression.^23^ Hemolysis was absent in the Δ*sly* strain, while the WT strain resulted in complete hemolysis across a wide range of dilutions (Figure 5A,B).

Transmission electron microscopy was used to visualize the morphological effects of the *cps* and *lgt* deletions on the *S. suis* capsule (Figure 5C,D). The polysaccharide capsule was clearly visible in wild-type P1/7 and in the Δ*lgt* mutant, whereas both Δ*cps* mutant strains lack the polysaccharide capsule. There was no clear morphological difference between the Δ*lgt* mutant strains and the corresponding parental strain expressing the *lgt* gene (data not shown).

In addition, we tested the activation of human TLR2/6 in transfected HEK-293 reporter cell lines by Δ*cps*, Δ*lgt*, and Δ*cps* Δ*lgt* strains (Figure 5E,F). These reporter cell lines were transfected with the pNiFty2 reporter plasmid, which expresses luciferase upon NF-κB signaling. As activation of TLRs leads to NF-κB signaling, the activation can be quantified by measuring the luminescent activity. A HEK-293 reporter cell line transfected with the pNiFty2 reporter plasmid but lacking TLRs was used as a control. The different HEK-293 cell lines were stimulated with each bacterial strain in penicillin-containing medium to release bacterial cell wall and membrane components.^24^

In the cell line expressing hTLR2/6, we observed a statistically significant difference (adjusted *p*-value = 0.0305) between luminescence upon stimulation with the Δ*cps* and Δ*lgt* strains (Figure 5E). Although the differences between the other samples did not reach statistical significance, a general trend could be observed. The deletion of *lgt* resulted in reduced TLR2/6 signaling, whereas the deletion of *cpsEF* increased TLR2/6 activation. When the reporter cells were stimulated with the Δ*cps* Δ*lgt* double mutant strain, we measured similar values as for the wildtype, suggesting that the opposing effects observed for the single gene mutants cancel each other out. No increased luminescence was measured in control HEK-293 cells that did not express TLRs for any tested condition, except for the positive control TNFα, which activates the NF-κB pathway independently of TLR signaling (Figure 5F). The results of the TLR activation assay are presented as luminescent activity relative to the values measured for the unstimulated baseline controls of the same cell line.

Our findings correspond to earlier research on the function of these genes. The main ligand leading to activation of TLR2/6 is diacetylated lipoproteins.^25^ Because the function of the *lgt* gene is to catalyze the transfer of a diacylglyceryl group to prelipoproteins,^26^ the deletion results in the absence of the immunogenic diacylglyceryl groups on prelipoproteins. One well-known function of capsular polysaccharide is to mask the bacterial surface and its associated, potentially immunogenic, lipoproteins to avoid recognition by the immune system.^27^ Hence, deletion of the *cpsEF* genes results in more exposed pathogen-associated molecular patterns, such as diacetylated lipoproteins.

### Introduction of Single Amino Acid Changes in Essential Proteins by CRISPR-Cas9 Genome Editing

**Figure 6:**
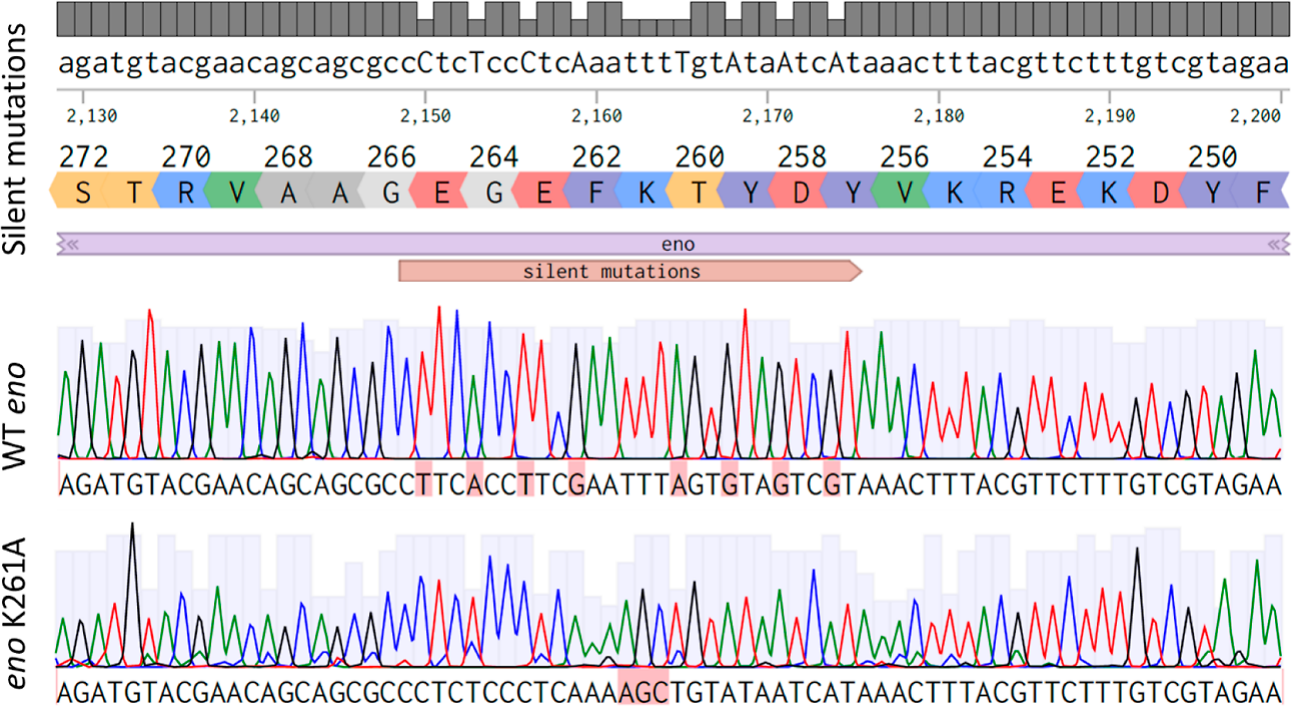
Alignment of Sanger sequencing reads showing the WT sequence and *eno*^*K261A*^. The top panel shows the WT sequence modified in silico by introducing silent mutations that do not affect the amino acid sequence. The middle panel shows Sanger sequencing results of the WT sequence, with the bases highlighted (pink boxes) that were changed to introduce the silent mutations displayed in the upper panel. The bottom panel shows the sequencing read corresponding to the successfully constructed mutant P1/7 *eno*^*K261A*^; the boxed sequence AGC indicates the K261A replacement.

To demonstrate the use of this system to study essential genes, we constructed a strain with a single amino acid change in the essential enolase (*eno*) gene. We designed a synthetic DNA fragment containing nonsynonymous nucleotide changes and additional silent mutations nearby so that no CRISPR targeting would be possible after HR. The original sgRNA designed for the target region, eno2, was not effective in killing the WT, which might be due to a sequence-specific effect such as, for example, a low GC content. Therefore, we modified the RT by introducing additional silent mutations approximately 500 bp downstream of the original target site, in a position where a highly efficient sgRNA was predicted by Benchling. The new sgRNA enabled efficient counter-selection against WT cells, resulting in strain P1/7 *eno*^*K261A*^ (Figure 6). To confirm the precision of the genome editing method presented here, we also sequenced the complete genome of P1/7 *eno*^*K261A*^ using the ONT long-read sequencing. Compared to the parental strain, no structural variations and no mutations other than the intended ones were found, highlighting the high accuracy of our genome editing method.

### Characterization of Small Colony Variants and “CRISPR-Escape” Mutants

To better understand the mechanisms behind the occurrence of SCVs, we restreaked them multiple times on selective and nonselective agar media. *S. suis* transformed with the nontargeting plasmid pSStarget-NT was used as a control. While SCVs retained their phenotype on antibiotic-selective media, they grew as normal-sized (large) colonies without antibiotic selection, suggesting the loss of the plasmid. After several passages on selective media, individual large colonies appeared among the SCVs, suggesting the occurrence of a mutation in the target site.

**Figure 7:**
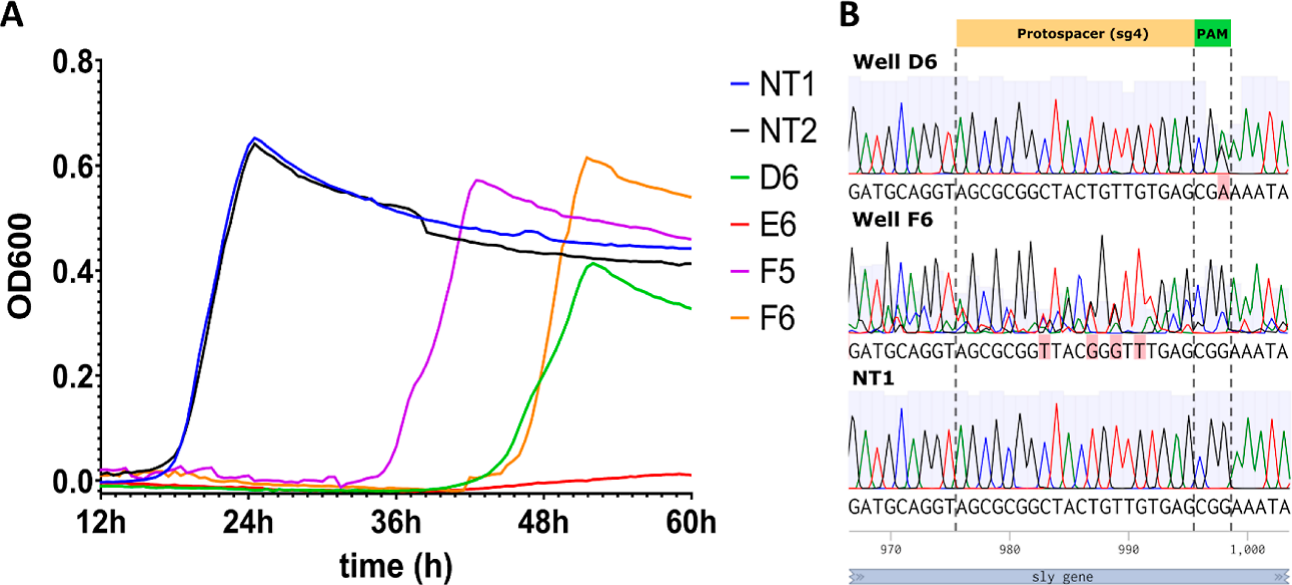
Growth of small-colony variants. (A) Growth curves recorded over 60 h in wells inoculated with colonies transformed with the nontargeting control pSStarget-NT (NT1, NT2) or SCVs recovered after transformation with pSStarget-sg4 (D6, E6, F5, and F6). While the control cultures displayed normal growth to maximum density over 24 h, the growth of SCVs was arrested or delayed (as shown in D6, E6, F5, and F6). (B) Sequencing of the target site revealed single-nucleotide substitutions (highlighted in pink) occurring in the protospacer (Well F6; 983C > T, 987T > G, 989T > G, and 901G > T) or PAM site (well D6, 998 G > A) of the sequence. No mutations were found in the NT control. The numbers below the nucleotide sequences refer to the nucleotide position in the *sly* coding sequence.

To analyze the growth phenotype of the SCVs, we measured growth in selective THY medium inoculated with SCVs recovered after transformation with pSStarget-sg4 (targeting *sly*) or the NT control. Colonies of bacteria transformed with NT control displayed normal growth, while cultures inoculated with small colonies did not grow after 24 h (Figure 7A). However, after incubation for 36–48 h, growth was observed in three of the eight wells (i.e., D6, F5, and F6) inoculated with SCVs (Figure 7A). Plating the cultures where growth was observed on selective agar media produced exclusively large colonies, while plating the medium from nongrowing colonies (e.g., E6) gave a small colony phenotype on agar plates. Sanger sequencing of the *sly* gene in D6, F5, and F6 revealed “CRISPR-escape” mutations in the target sites or adjacent PAM sequences, which would prevent Cas9-mediated DNA cleavage (Figure 7B). Bacteria in F6 acquired four mutations in the protospacer of the target site (983C > T, 987T > G, 989T > G, and 901G > T), which allowed normal growth under antibiotic selection for pSStarget-sg4. Bacteria in well D6 acquired a single-nucleotide substitution in the PAM site, changing the PAM sequence from CGG to CGA. Interestingly, well D6 had a lower growth rate during the exponential phase and reached a lower maximal density (Figure 7A, green curve). These results suggest that the SCVs might resemble persister cells, as the growth of the respective cells is temporarily arrested in response to stress.

**Figure 8:**
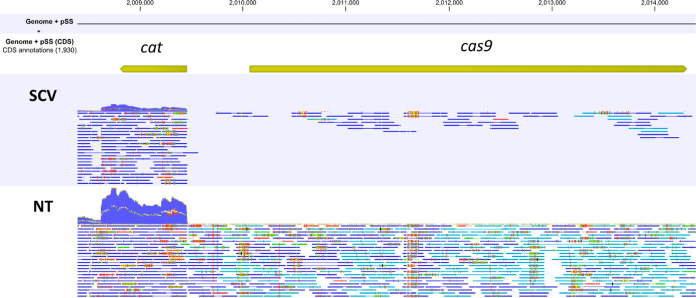
Mapping of RNaseq reads to the region of pSStarget containing the *cat* and *cas9* genes. The top panel shows the location of the *cat* and *cas9* genes. The two lower panels show the mapping of RNaseq reads to these two genes in the targeting (SCV) and nontargeting (NT) conditions, respectively. As in the case of the *cat* gene, when too many reads were present to plot each read individually, the reads were visualized as a pileup plot. Both genes were strongly downregulated in the SCV condition (upper panel) compared to the NT condition (lower panel)

To gain further insights into the mechanism of CRISPR-tolerance in SCVs, we conducted a transcriptomic analysis comparing gene expression in SCVs recovered after transformation with pSStarget-sg6 (SCV) to the gene expression in bacteria recovered after transformation with pSStarget-NT (NT), lacking a targeting spacer as a control. At the time of sample collection, we plated an aliquot of each culture to ensure that the small colony phenotype had been maintained. Mapping of the RNaseq reads to the plasmid sequence verified the expression of all critical plasmid components in the NT group, including the sgRNA expression cassette (Figure S4). We found that all plasmid-encoded genes were strongly downregulated (50-fold to 113-fold reduction) in the SCVs compared to the NT control, suggesting a reduction in the plasmid copy number. The most downregulated gene was the chloramphenicol (Cml) resistance marker (*cat*) (113-fold reduction), suggesting growth inhibition due to the bacteriostatic effect of Cml. Moreover, the *cas9* gene was downregulated 51-fold, which may not be sufficient to induce DNA cleavage (Figure 8). This observation led us to hypothesize that SCV formation might depend on maintaining a balance between sufficient *cat* expression to maintain Cml resistance and a low level of *cas9* expression. Indeed, plating the transformed bacteria on agar containing an increased Cml concentration of 7.5 μg/mL resulted in a clear reduction of SCVs relative to the usual Cml concentration of 5 μg/mL (Figure S5).

Other downregulated genes included growth-related genes such as the *fab* and *acc* operons (SSU1597–1609) which are involved in lipid biosynthesis, genes encoding enzymes from the tricarboxylic acid cycle (SSU1040–SSU1042), and genes involved in glycolysis (Table 1). The most upregulated genes in the SCVs included genes involved in pyrimidine metabolism (SSU0735–SSU0738 and SSU0860–SSU0868) and genes involved in pentose and glucuronate interconversion (SSU0998-SSU1008). Additionally, multiple peptidoglycan-binding genes were upregulated, including two genes encoding murein hydrolases (SSU1854 and SSU1911) that are involved in competence-induced cell death (fratricide) in other streptococci. Four of the six chromosomally encoded toxin-antitoxin systems were upregulated in the SCVs (SSU0571/572, SSU1318/1319, SSU1795/1796, and SSU1798/1799). Furthermore, two genes involved in cell division (SSU0106 and SSU0859) and genes involved in DNA repair, including the mismatch repair proteins MutT and MutX, the uracil-DNA-glycosylase Ung (involved in base excision repair), and the recombinase RecF, had higher expression levels in the SCVs than in the nontargeting control. Also, the *smpB* gene (SSU1218), which is involved in rescue of stalled ribosomes, was upregulated. Finally, transport proteins, including a uracil permease (SSU1729), multiple putative drug export pumps (SSU0573, SSU0744, SSU0745, and SSU1242), three MFS transporters (SSU1181, SSU1555, and SSU1912), and a variety of ABC-transporters and PTS systems, were upregulated in the SCVs. The complete list of all up- and downregulated genes can be found in the Supporting Information Table S3.

**Table 1:** Log2 Fold-Change (FC) in Expression Values of Selected Differentially Expressed Genes (SCV vs NT)

locus tag	gene symbol	functions	Log2 FC
		plasmid-encoded genes	
N/A	*cas9*		–5.67
N/A	*Cat*		–6.82
N/A	*p15A*		–5.67
N/A	*repD*		–6.45
N/A	*tetA*		–5.71
		lipid biosynthesis	
**SSU1597**	*accA*		–5.42
**SSU1598**	*accD*		–5.45
**SSU1599**	*accC*		–5.81
**SSU1600**	*fabZ*		–5.99
**SSU1601**	*fabE*		–6.15
**SSU1602**	*fabF*		–6.29
**SSU1603**	*fabG*		–6.65
**SSU1604**	*fabD*		–6.56
**SSU1605**	*fabK*		–6.50
**SSU1606**	*acpP*		–4.67
**SSU1607**	*fabH*		–4.05
**SSU1608**	*marR*		–3.78
**SSU1609**	*phaB*		–5.05
		tircarboxylic acid cycle	
**SSU1040**	*Icd*		–2.98
**SSU1041**	*citZ*		–2.92
**SSU1042**	*acnA*		–2.93
		glycolysis	
**SSU0153**	*Plr*		–1.17
**SSU0154**	*Pgk*		–1.28
**SSU0312**	*Fba*		–1.56
**SSU0483**	*Tpi*		–1.12
**SSU0927**	*Ldh*		–1.34
**SSU1308**	*bglH*		–1.41
**SSU1309**			–1.54
**SSU1320**	*Eno*		–1.46
**SSU1451**	*gpmA*		–1.47
**SSU1836**	*Pgi*		–1.07
		pyrimidine metabolism	
**SSU0735**	*pyrR*		2.55
**SSU0736**	*pyrB*		2.45
**SSU0737**	*carA*		2.54
**SSU0738**	*carB*		2.64
**SSU0860**	*sntC*		1.23
**SSU0861**	*pyrC*		2.41
**SSU0863**	*yfdH*		2.27
**SSU0864**	*pyre*		3.40
**SSU0865**			3.74
**SSU0866**	*pyrF*		3.81
**SSU0867**	*pyrD*		3.52
**SSU0868**	*pyrK*		3.63
		pentose and glucuronate interconversion	
**SSU0998**	*nag3*		2.56
**SSU0999**			2.47
**SSU1000**			2.52
**SSU1001**	*kduD*		2.33
**SSU1002**	*uxuA*		2.02
**SSU1003**	*uxaC*		2.24
**SSU1006**	*uidA*		2.74
**SSU1007**	*kdgK*		2.71
**SSU1008**			2.33
		peptidoglycan binding	
**SSU0020**	*pcsB*		1.72
**SSU0215**	*Sip*		2.09
**SSU1854**	*lytF*		4.04
**SSU1911**	*cbpD*		1.39
**SSU1950**	*lytE*		2.92
		toxin-antitoxin systems	
**SSU0571**			1.39
**SSU0572**			0.96
**SSU1318**			1.87
**SSU1319**			1.96
**SSU1795**	*yoeB*		1.42
**SSU1796**			1.48
**SSU1798**			1.29
**SSU1799**			1.45
		DNA translocase (cell division)	
**SSU0106**	*ftsK*		1.34
		septum formation inhibitor (cell division)	
**SSU0859**	*Maf*		1.66
		DNA repair	
**SSU0251**	*mutT*		1.35
**SSU0862**	*Ung*		2.34
**SSU1763**	*mutT*		1.30
**SSU1797**	*mutX*		1.12
**SSU1959**	*recF*		1.04
		rescue of stalled ribosomes	
**SSU1218**	*smpB*		1.31
		transport proteins	
**SSU0573**	*norm*		1.06
**SSU0744**	*msbA_1*		1.53
**SSU0745**	*lmrA*		1.52
**SSU1181**	*ywaF*		1.53
**SSU1242**	*vanZ*		1.63
**SSU1729**	*pyrP*		3.13
**SSU1912**	*MFS*		1.12

## Discussion

In recent years, several tools have been developed for the genetic engineering of *S. suis*, including the discovery of the competence-inducing peptide XIP and the development of combined selection-counterselection cassettes for marker removal.^10,12^ Recently, we have shown that strains of *S. suis* grown in active porcine or human serum become competent for DNA transformation.^28^ Although this method is less efficient than peptide-induced natural competence, it is simple compared to other methods and works with strains that contain different competence peptide alleles or which are not competent via the peptide-induced pathway.^28^

To enrich the toolbox for genetic manipulation of *S. suis* and enable efficient “markerless” genome editing, we developed a plasmid-based CRISPR-Cas9 system. The method involves transformation with the plasmid pSStarget that expresses Cas9 and a sgRNA in combination with a homologous RT. Our CRISPR-Cas9 system cleaves the targeted WT sequence and efficiently counter-selects against nonedited cells that contain the targeted WT sequence in the genome. The high efficiency of this system drastically reduces the number of colonies to be screened by colony PCR.

The pSStarget plasmid contains a pIL253 replicon, which is a high-copy number vector that replicates in a variety of Gram-positive bacteria, including lactococci, enterococci, and streptococci.^18,29–31^ Due to the broad host range of this vector, the system can likely be used in other organisms, pending minor adaptations where necessary. In *S. suis*, we showed that pIL253 is segregationally unstable in the absence of antibiotic selection and is easily cured by subculture in nonselective medium. A *ccdB* gene encoding the gyrase inhibitor CcdB, a potent toxic protein flanked by BsaI recognition sites, was introduced in the sgRNA cloning site. The *ccdB* gene counter-selects against maintenance of plasmids in common laboratory strains of *E. coli,*which are sensitive to killing by CcdB. Digestion of pSStarget with BsaI and ligation of the annealed spacer oligonucleotides into the plasmid replaces the *ccdB* gene. Transformation of the original (*ccdB*-containing) plasmid leads to production of the CcdB toxin, resulting in the death of such cells and thereby ensuring that all recovered colonies contain the desired sgRNA insert. pSStarget also contains the p15A replicon, which facilitates a quick and easy workflow for cloning and plasmid preparation in a CcdB-resistant strain of *E. coli*.

The segregationally unstable plasmid allows for multiple sequential rounds of genome editing. Subculture overnight in nonselective medium ensures a rapid loss of the plasmid and associated selection marker, allowing the introduction of a second plasmid on the following day. We also demonstrated the use of this CRISPR system to edit essential genes. The unprecedented accuracy of this genome editing system allows the introduction of single base pair mutations leading to amino acid changes in genes that are essential for growth in laboratory medium. This facilitates the study of essential genes by modification of different parts and domains of the encoded protein.

In accordance with previous reports of CRISPR-mediated genome editing in bacteria, we found a few colonies that survived CRISPR selection without incorporation of the RT. Sequence analysis of such colonies showed random inactivating mutations in the target site or CRISPR system.^18,32^ We also discovered a different novel mechanism by which *S. suis* can escape killing by a targeted Cas9-sgRNA complex in the absence of a homologous RT. A visual characteristic of this novel mechanism is the presence of very small colonies (SCVs) after transformation and plating. These small colonies grew extremely slowly in liquid culture, typical of a dormant, persister-like state. However, after 36 to 48 h, some isolates resumed growth due to mutations in the target sequence for the sgRNA or PAM site (Figure 7), suggesting that slow growth may have allowed for DNA repair or the introduction of mutations interfering with lethal Cas9 nuclease activity. When growth resumed, two cultures followed a growth pattern similar to that of the NT control, while a third culture (D6) appeared to have a lower growth rate and final density. Sequencing of target sites revealed a single-nucleotide substitution in the PAM site that had altered the corresponding PAM sequence from CGG to CGA. Previous reports have shown that *S. pyogenes* (Sp) Cas9 can recognize and cleave targets flanked by a NGA PAM with efficiencies of up to 15% compared to targets flanked by a canonical NGG PAM,^33,34^ suggesting that the observed differences in growth phenotype of culture D6 could be a consequence of the residual Cas9-activity, leading to the death of a subset of cells. The number of SCVs was highly dependent on the sgRNA sequence. While pSStarget-sg4 resulted in very few small colonies, transformation with pSStarget-sg6 yielded substantially more small colonies. Both sgRNAs had a comparable on-target score (66.5 vs 65.5) but differed substantially in GC content (60 vs 20%). This suggests that the on-target score should not be used as the only metric for sgRNA selection but that additional criteria, such as GC content, should be taken into consideration during sgRNA selection.

RNaseq analysis of SCVs revealed that all pSStarget plasmid-encoded genes were strongly downregulated, suggesting that the plasmid copy number had been reduced. This reduction of pSStarget copy number may have reduced Cas9-induced stress and resistance to Chloramphenicol (Cml), thereby affecting growth and colony size. We found a variety of multidrug efflux pumps and other transport proteins up-regulated in the SCVs, which could contribute to lowering intracellular Cml concentrations. A transcriptomic analysis of *Bacillus subtilis* exposed to subinhibitory concentrations of Cml showed an upregulation of multiple transport proteins, including a multidrug efflux pump and the uracil permease *pyrP*(35) together with upregulation of the pyrimidine biosynthesis gene cluster, which were upregulated in our experiments as well.

Furthermore, we saw the induction of four toxin-antitoxin (TA) systems, members of the *relEB* and *yoeB/yefM* families, that inhibit translation via cleavage of translated mRNAs, leading to nonstop mRNAs and stalling of ribosomes. In line with this, we observed a 3-fold upregulation of the *smpB* gene, which plays a crucial role in the rescue of stalled ribosomes via trans–translation.^36^ We also noted upregulation of the gene encoding RNase R that is recruited to the rescue of stalled ribosomes for specific degradation of nonstop mRNA.^37,38^ Induction of TA systems during stress conditions has been linked to persister cell formation.^39–41^ Specifically, the RelE-type toxins induce a transient bacteriostatic state through potent inhibition of the translation machinery, and induction of the corresponding antitoxin rapidly reverts the inhibition phenotype.^42^ In the pathogenic bacteria *Edwardsiella piscicida*, a homologue of *yoeB* (SSU1795) has been shown to increase resistance to chloramphenicol and other antibiotics and plays an important role in persister cell formation at lethal chloramphenicol concentrations.^43^ Induction of chromosomal TA systems could have led to the dormant state observed for SCVs in our experiments.

Translational inhibition and reduced plasmid copy numbers could underpin the mechanisms of CRISPR-escape. Cas9 is a large protein (1371 AA) and its production would likely be more affected by translational inhibition compared to that of smaller proteins. Combined with a 50-fold reduction in mRNA expression, it is likely that very few, if any, functional Cas9 proteins can be produced in this condition, preventing the lethal nuclease activity.

Overall, the findings of our transcriptome analysis of SCVs are consistent with the observed growth inhibition. Downregulation of specific genes involved in lipid biosynthesis, the TCA cycle, and glycolysis corresponds to reduced metabolism and growth rate and underpins a state of dormancy. A state of reduced metabolic activity and growth rate has been linked to increased antibiotic tolerance and persister cell formation in *Staphylococcus aureus*.^44^ Genes involved in DNA repair and recombination have been upregulated in and a cold-shock protein (SSU0368), that was shown to be involved in compensating for a mutator phenotype, was downregulated in the SCVs.^45,46^ In combination with the results from the prolonged growth curve experiment, it is intriguing to suggest that the induced mechanism of dormancy allowed mutations in the targeted DNA site to accumulate, leading to escape from the CRISPR endonuclease activity and reversion to the normal growth phenotype.

The insights into the mechanisms underlying SCV formation were used to improve the efficiency of the editing process. For example, sgRNA targets with a low GC content seem to lead to more SCV formation and should be avoided when possible. To account for the variable sgRNA efficiency, it can be useful to include a “background control” by transforming only the pSStarget plasmid without the corresponding RT. A further recommendation is to maintain chloramphenicol (Cml) selection pressure for a few passages to avoid the growth of nonedited SCVs. This strategy is especially useful in cases where only low-GC-content targets are available for sgRNA design, and thus the formation of many SCVs in addition to larger (normal-sized) colonies can be expected, making it more difficult to select a single larger colony. Another approach could be to adjust the concentration of Cml to reduce the formation of SCVs. We theorize that SCVs are formed by lowering the copy number of the pSStarget plasmid (or expression levels of the encoded genes) to allow sufficient *cat* expression to grow slowly in the presence of Cml stress but not sufficient expression of the *cas9* gene to induce a double-stranded break in the genomic DNA by Cas9. When the Cml concentration is increased from 5 to 7.5 μg/mL, the SCV formation is greatly reduced in strain P1/7. The optimal concentration of Cml required to suppress SCV formation is likely to be strain-dependent and might need to be optimized when this system is adaptable for use in other strains.

## Materials and Methods

### Growth of Bacterial Strains

All strains used in this study are listed in Table 2. Liquid cultures of *S. suis* were grown in Todd-Hewitt broth (Oxoid) supplemented with 0.2% Bacto yeast extract (BD Biosciences) (THY) without agitation. For hemolysis assays, a complex medium supplemented with 1% (w/v) Pullulan (Sigma-Aldrich) (CM + Pul) has been prepared as previously described.^47^ For agar plate cultures, THY supplemented with 1.5% agar (Thermo Fisher) or Columbia agar plates (Thermo Fisher) supplemented with 5% sheep blood (Thermo Fisher) were used. Cultures were incubated in a humidified incubator at 37 °C and a 5% CO_2_ level. Unless otherwise noted, chloramphenicol (Sigma-Aldrich) was added to a concentration of 5 μg/mL.

**Table 2:** Bacterial Strains Used in This Study

strain	relevant characteristics	refs
E. coli ccdB survival 2	*ccdB*-resistant cloning host	Invitrogen
E. coli NEBturbo	*ccdB*-sensitive cloning host	New England Biolabs
S. suis P1/7	serotype 2 type strain	Laboratory collection
S. suis P1/7 Δ*sly*	*sly* knockout mutant	this work
S. suis P1/7 Δ*cps*	*cps* knockout mutant	this work
S. suis P1/7 Δ*lgt*	*lgt* knockout mutant	this work
S. suis P1/7 Δ*cps* Δ*sly*	*cps* + *sly* double-knockout mutant	this work
S. suis P1/7 Δ*cps* Δ*lgt*	*cps* + *lgt* double-knockout mutant	this work
S. suis P1/7 *eno*^*K261A*^	single amino acid substitution in *eno* (K261A)	this work

*E. coli*NEBturbo was used as a general cloning host. *E. coli*was cultured in a Luria–Bertani (LB) medium (Merck) for liquid cultures. For agar plate cultures, LB was supplemented with 1.5% agar. When appropriate, antibiotics were added to the medium at following concentrations: chloramphenicol (Cml) 25 μg/mL; kanamycin (Merck) (Kan) 50 μg/mL; and tetracycline (Sigma-Aldrich) (Tet) 10 μg/mL. The cultures were incubated at 37 °C in a dry incubator without adding CO_2_. Liquid cultures were grown in a shaking incubator at 250 rpm.

### General DNA Manipulations and Transformation

All plasmids, oligonucleotides, and sgRNAs used in this study are listed in Table 3 and Supporting Information Tables S1 and S2, respectively. Plasmid DNA was routinely isolated from *E. coli*cultures using the QIAprep Spin Miniprep Kit (Qiagen). When large amounts of plasmid were required, the Plasmid Plus Midi Kit (Qiagen) was used for purification. Cloning PCRs were performed using Q5 High-Fidelity DNA Polymerase (New England Biolabs). Oligonucleotides were synthesized by IDT. PCR products were purified using the MSB Spin PCRapace kit (Invitek). For the isolation of Genomic DNA (gDNA), bacterial cells were transferred to a tube containing 0.1 mm silica beads (MP Biomedicals) and lysed by bead beating for 40 s at 4.0 m/s using a FastPrep-24 5G (MP Biomedicals). The lysates were cleared by centrifugation 16.000× for 10 min, and the gDNA has been isolated using the PowerSoil DNA Isolation Kit (Qiagen) according to manufacturer’s instructions. Sanger sequencing was performed using the LightRun sequencing service according to instructions (Eurofins Genomics).

**Table 3:** Plasmids Used in This Study

plasmid	relevant characteristics	refs
pACYC184	contains p15A ori and *tetA*	ATCC
pDONR221	contains *ccdB* gene	Invitrogen
pLABtargetc	CRISPR-Cas9 vector developed for *Lactobacilli*	(18)
pSStarget-NT	pLABTargetc derivative containing the p15A ori and a *tetA* resistance gene, no sgRNA spacer sequence	this work
pSStarget	pSStarget-NT derivative containing *ccdB* stuffer region in the sgRNA cloning site	this work
pSStarget-sg4	pSStarget with sg4 inserted (targeting *sly*)	this work
pSStarget-sg6	pSStarget with sg6 inserted (targeting *cps*)	this work
pSStarget-sg42	pSStarget with sg42 inserted (targeting *lgt*)	this work
pSStarget-eno2	pSStarget with sg_eno2 inserted (targeting *eno*)	this work
pSStarget-eno3	pSStarget with sg_eno3 inserted (targeting *eno*)	this work

Chemically competent *E. coli*strains were transformed by heat shock. *S. suis* strains were transformed as previously described by inducing the natural competence pathway via the addition of the XIP peptide.^10^ Briefly, *S. suis* overnight cultures were diluted 40-fold and grown to an OD_600_ of approximately 0.04–0.05. Aliquots of 100 μL of bacterial culture were transferred to a sterile microcentrifuge tube, and 1–10 μg of transforming DNA was added to each tube. The XIP peptide (Genscript) was added to a final concentration of 250 μM, and the cultures were incubated for 2 h at 37 °C with 5% CO_2_ before plating 100 μL of transformation mix on selective agar plates. Appropriate dilutions were plated to allow the selection of single colonies.

### Construction of pSStarget

For the construction of pSStarget, we used the previously published pLABtargetc as a starting point.^18^ To enable replication of this vector in *E. coli*, a fragment containing the p15A origin of replication (ori) and the tetracycline resistance gene *tetA* was amplified from pACYC184 (ATCC) using primers P1/P2. The pLABtargetc was linearized using *Sma*I (Thermo Fisher), and the purified PCR product was cloned into the linearized vector by blunt cloning using T4 Ligase (Promega) and transformed to *E. coli*Top 10 competent cells, resulting in vector pSStarget-NT. Next, pSStarget-NT was digested using BsaI-HFv2 (New England Biolabs). The *ccdB* region was amplified from pDONR221 (Invitrogen) using primers P3/P4. The purified fragments were assembled using the NEBuilder HiFi DNA Assembly Kit (New England Biolabs) and transformed to *E. coli*One Shot ccdB Survival 2 T1R Competent Cells (Invitrogen). The final plasmid was sequence-verified using the certified Oxford Nanopore Technology (ONT) plasmid sequencing service offered by Plasmidsaurus.

### Design and Assembly of sgRNAs in pSStarget

The sgRNAs were designed using the CRISPR tool in Benchling.^48^ The desired target region was selected, and the computed sgRNAs were sorted according to their on-target score.^49^ Potential off-target sites in the *S. suis* P1/7 genome were predicted using Cas-Designer.^50^ For each target gene, multiple sgRNAs with the highest on-target scores were selected, and sgRNAs with potential off-target sites were omitted. To allow cloning into pSStarget, the nucleotide sequence “TGAT” was appended upstream of the target-specific sgRNA sequence, and “GTTT” was appended downstream of that sequence. In cases in which the sgRNA did not start with a G, the sequence “TGATG” was appended upstream instead, ensuring that each sgRNA started with a G. This sequence was used to design partially complementary oligonucleotides, which result in a dsDNA product with 4 bp overhangs on each side upon annealing that are complementary to the overhangs created on the pSStarget vector upon BsaI digestion.

The complementary oligonucleotides were mixed in annealing buffer (10 mM Tris pH 8, 50 mM NaCl) to yield a final concentration of 10 μM for each oligonucleotide. For annealing, this mixture was heated to 95 °C for 2 min and gradually cooled down to 25 °C over the course of 45 min. The resulting dsDNA fragments were cloned into pSStarget in a Golden Gate-like reaction. Each reaction contained 75 ng of pSStarget, 1 μL of annealed oligonucleotides (sgRNA), 2.5 μL of T4 DNA ligase buffer (10×), 1000 units of T4 DNA ligase (NEB#M0202, New England Biolabs), and 30 units of BsaI-HFv2 (NEB#R3733, New England Biolabs) in a total volume of 25 μL. This assembly mixture was then incubated in a thermocycler for 60 cycles (37 °C for 5 min, 16 °C for 5 min), followed by an inactivation step for 5 min at 65 °C. The reaction mixtures were then transformed to competent *E. coli*NEBturbo.

### Construction of Homologous Repair Templates

Homologous RTs were assembled by SOEing (Splicing by Overlap Extension) PCR.^51^ For each deletion, flanking regions of approximately 1000 bp were selected directly upstream (US) and downstream (DS) of the region to be deleted. The NEBuilder assembly tool was used for primer design with matching overlaps on the internal primers, using a minimal overlap of 20 nt and unchecking the “circularize” box.^52^ The US and DS fragments were amplified by PCR using P1/7 gDNA as a template. The purified fragments were mixed in equimolar amounts and joined in a PCR lacking primers. The reaction products were diluted 50-fold and amplified by PCR using distal primers (US_Fw/DS_Rv).

For site-directed mutagenesis of the *eno* gene, a circular assembly was made, including a synthetic fragment containing the desired mutations (GeneArt), the homologous flanks, and a pUC57-kan (Genscript) fragment encoding kanamycin resistance and a replicon. All primers (P27–P32) were designed using the NEBuilder assembly tool, and the fragments were PCR-amplified. The purified PCR products were assembled using HiFi assembly (New England Biolabs), and the linear RT was amplified by PCR from the assembly mixture using primers P33/P34. Using this fragment as a template, two fragments of approximately 1500 and 500 nt were amplified using primers P33/P36 and P34/P35, thereby introducing silent mutations in the 5′ primer overhangs. The fragments were purified and joined by SOEing as described above, yielding the final RT used for the *S. suis* transformation.

### Mutagenesis Using CRISPR-Cas9

*S. suis* mutant strains were generated by simultaneously cotransforming a homologous RT and a pSStarget plasmid containing an sgRNA complementary to the target region. The transformants were plated on agar plates containing chloramphenicol to select for cells containing pSStarget. Selected colonies were restreaked on selective plates and tested by colony PCR using the corresponding distal primers for the presence of a band corresponding to the length of the RT.

### DNA Sequencing and Data Analysis

Short-read genome sequencing was provided by MicrobesNG (https.microbesng.uk). *S. suis* P1/7 and KO strains were grown overnight on THY agar plates. All of the growth was collected with a sterile loop and resuspended in MicrobesNG bead tubes containing cryopreservant solution. The vial contents were mixed and sent to MicrobesNG for DNA extraction, library preparation, and Illumina sequencing. The raw reads were analyzed by the MicrobesNG pipeline and mapped against the P1/7 reference sequence (GenBank: AM946016.1) to construct a consensus sequence. Long-read sequencing using ONT was performed by Plasmidsaurus. Briefly, genomic DNA was minimally fragmented, amplification-free libraries were constructed from the fragmented DNA using the v14 library prep chemistry, and the libraries were sequenced using R10.4.1 flow cells (ONT). The data was processed by Plasmidsaurus to produce high-quality consensus sequences using Flye for assembly and medaka for polishing.

Further data analysis has been performed using tools hosted by the public server (usegalaxy.eu) of the European Galaxy Project.^53^ To detect SNPs and InDel mutations, the consensus sequences were compared to the reference sequence by using the diffseq tool. To detect larger structural rearrangements, the raw ONT reads were mapped against the reference sequence, and the resulting alignments were sorted by genomic coordinates using Samtools sort with activated minash (−M) collation. Structural rearrangements relative to the reference sequence were detected using cuteSV. Reference-based consensus sequences were derived from the mapped reads using the iVar consensus caller. The raw sequencing data is publicly available at the 4TU.ResearchData repository.^54,55^

### Hemolysis Assay

The hemolysis assay has been conducted in triplicate as previously described with minor modifications.^56^ Briefly, *S. suis* strains were grown overnight in CM + Pul. The supernatants from 1 mL of culture were collected by centrifugation, and 100 μL of the supernatants were transferred to 96-well round-bottom microtiter plates. Subsequently, serial 2-fold dilutions were prepared in sterile phosphate buffered saline (PanReac AppliChem) (PBS). A solution containing 2% sheep blood (Thermo Fisher) and 0.5% bovine serum albumin (Sigma-Aldrich) has been prepared in PBS, and 100 μL of this solution was added to each well. The plates were incubated at 37 °C for 2 h, after which the nonlysed erythrocytes were sedimented by centrifugation at 1500*g* for 10 min. The lysed erythrocytes were quantified by measuring absorption at 540 nm. A 1% Triton-X100 solution (Merck) has been used as a reference for complete lysis.

### Electron Microscopy

Electron microscopy was used to verify capsule loss in the Δ*cps* mutant, as previously described.^57^ Briefly, strain P1/7 and the derived mutant strains were grown in liquid culture to the mid logarithmic phase, and the cells were collected by centrifugation for 10 min at 8000*g*. The pellet was fixed according to the lysine-acetate-based formaldehyde/glutaraldehyde ruthenium red-osmium fixation procedure, which involves fixation in a series of three fixatives containing combinations of 2% formaldehyde, 2.5% glutaraldehyde, 100 mM cacodylate buffer, 0.075% ruthenium red (RR), 75 mM lysine acetate, and 1% osmium tetroxide. After each fixation step, the samples were washed in 100 mM cacodylate buffer with 0.075% RR, and a second wash was performed after the final fixation step. The samples were dehydrated using a series of increasing ethanol concentrations and embedded in Epon. The prepared samples were analyzed using the JEM-1400 Plus transmission electron microscope (JEOL) with a voltage of 80 kV.

### Stimulation of HEK-293 Cells Expressing Human TLR1/2 and TLR2/6

To quantify human Toll-like receptor (hTLR) activation by *S. suis* P1/7 and the mutant strains, we used human embryonic kidney (HEK) cells transfected with the pNiFty2 luciferase reporter plasmid (InvivoGen). Two different pNiFty2 reporter cell lines were used: a cell line expressing hTLR2 and hTLR6, and a control cell line which did not express any TLRs. The cells were maintained using Dulbecco’s modified Eagle’s medium, GlutaMAX + glucose (Gibco), supplemented with 10% Fetal calf serum (Gibco) (FCS) and 5% Pen-Strep solution (Sigma). When appropriate, the following antibiotics were added for selection and infection prevention: zeocin (InvivoGen) (250 μg/mL), blasticidin (InvivoGen) (10 μg/mL), puromycin (InvivoGen) (2 μg/mL), and normocin (InvivoGen) (100 μg/mL).

The two HEK-cell lines were seeded (6 × 10^4^ cells/well) into a black 96-well plate with a clear bottom and incubated overnight at 37 °C with 5% CO_2_. The following day, the medium was replaced with a new medium containing 30 μg/mL Penicillin (Sigma-Aldrich). The penicillin was added to stimulate the release of components that are firmly attached to the bacterial cell wall or membrane.^24^ The cells were stimulated with 2.0 × 10^7^ bacteria/mL for 3 h at 37 °C with 5% CO_2_. As positive controls, TNFα (Invitrogen, 100 ng/mL) and PAM2CSK4 (Invitrogen, 20 ng/mL) were used. Flagellin (Invitrogen, 500 ng/mL) was used as a negative control, and unstimulated samples were used as baseline controls. After the incubation period, the medium was replaced by 120 μL of Bright Glo luciferase substrate (Promega) and vortexed at 500 rpm for 5 min before the luminescence was measured using a SpectraMax M5 microplate reader (750 ms integration time). The results were expressed relative to the unstimulated control of the same cell line, with the values for the unstimulated control set to 1.

### Transcriptomic Analysis

Bacterial colonies were collected from an agar plate using a sterile loop and resuspended in 1 mL of THY containing Cml to a density (OD_600_) of 1. The bacterial suspensions were used to inoculate 6 mL of THY + Cml in triplicate at a starting OD_600_ of 0.06. The cultures were grown for 2.5 h at 37 °C with 5% CO_2_, and the bacteria were collected by centrifugation. The pellet was resuspended in QIAzol lysis reagent (Qiagen), transferred to a tube containing 0.1 mm silica beads (MP Biomedicals), and lysed by bead beating for 40 s at 4.0 m/s using a FastPrep-24 5G (MP Biomedicals). The lysates were cleared by centrifugation at 16.000× for 10 min. RNA isolation from the supernatants has been performed using the miRNeasy mini kit (Qiagen) according to manufacturer’s instructions. The quantity and quality of the purified RNA were determined using the Qubit 4 Fluorometer (Thermo Fisher) and the DS-11 UV–vis spectrophotometer (DeNovix). The RNA integrity was quantified using the 2200 TapeStation electrophoresis system (Agilent). The Illumina library preparation and sequencing using the Hiseq Illumina PE150 platform have been performed by Novogene Bioinformatics Technology Co., Ltd. in Hong Kong.

The data analysis has been conducted using CLC Genomics Workbench 20 (Qiagen). FASTQ reads were mapped against a custom-built reference sequence consisting of the *S. suis* P1/7 genome (NCBI accession: AM946016) concatenated with the sequence of pSStarget-NT. The expression values were normalized using transcripts per million (TPM), and a two-tailed *t*-test with adjusted *p*-values (FDR) was used to compare the gene expression between samples. A complete list of all differentially expressed genes can be found in Table S3. The raw data is available for download at the 4TU.ResearchData.^58^

## Data Availability

The sequence of the pSStarget plasmid is available on GenBank (accession number: OQ971745). The sequencing data generated in this study is openly available at the 4TU. ResearchData data repository and can be accessed using the following DOIs: DOI:10.4121/21679886 for the Illumina short-read genome sequencing data; DOI:10.4121/6affb4a6-2893-41e8-bdf8-d276247d7d19 for the Oxford Nanopore long-read sequencing data; and DOI:10.4121/21680384 for the RNaseq data.

## Supporting Information Available

Overview of all oligonucleotides used in this study, including sequence and short description; table containing an overview of sequences and calculated properties of all sgRNA spacers used in this study; table summarizing the fold-change in expression values of all differentially expressed genes in SCVs compared to NT control, the first sheet contains a general description of the data set and author information, the second sheet (all_diff_expressed) contains all differentially expressed genes together with the associated fold-changes and *p*-values, and the third (down_diff_expressed) and fourth (up_diff_expressed) sheet data sets have been split up in genes that were downregulated and upregulated in the SCVs, respectively; schematic representation of RNaseq data mapped to the sequence of pSStarget-NT, closeup view on the region encoding the sgRNA (nt: 6292–6429) shows reads mapped to the entire length of the sgRNA cassette demonstrating expression of this locus; and representative pictures of colony morphologies observed after plating transformation mix on CBA supplemented with 5% sheep blood and an increased Chloramphenicol concentration of 7.5 μg/mL (PDF)
Supporting Table 3. Differentially expressed genes in SCVs compared to NT control. (XLSX)
