# Supplementary material for: Precision Genome Engineering in Streptococcus suis Based on a Broad-Host-Range Vector and CRISPR-Cas9 Technology
Source: ACS Synth Biol. 2023 Aug 21;12(9):2546–60. doi: 10.1021/acssynbio.3c00110 (PMC10510748; doi:10.1021/acssynbio.3c00110)
Supplement: Supplementary file 1 — sb3c00110_si_001.pdf [file sb3c00110_si_001.pdf]

Supporting Information

Precision genome engineering in *Streptococcus suis* based on a broad-host-range vector and CRISPR-Cas9 technology

Authors: Alex Gussak[1], Maria Laura Ferrando[1], Mels Schrama[1], Peter van Baarlen[1], Jerry Mark Wells[1] *

[1] Host-Microbe Interactomics, Animal Sciences, Wageningen University, 6708 WD Wageningen, The Netherlands.

*Corresponding author, Email: jerry.wells@wur.nl

Supporting Table S1: Overview of all oligonucleotides used in this study including sequence and short description.

Supporting Table S2: Table containing an overview of sequences and calculated properties of all sgRNA spacers used in this study.

Supporting Table S3 (in separate .xlsx file): Table summarizing the Fold-change in expression values of all differentially expressed genes in SCVs compared to NT control. The first sheet of the .xlsx file contains a general description of the dataset and author information. The second sheet (all_diff_expressed) contains all differentially expressed genes together with the associated fold-changes (FC) and p-values. In the third (down_diff_expressed) and fourth (up_diff_expressed) sheets, the same dataset has been split up in genes that were downregulated and upregulated in the SCVs, respectively.

Supporting Figure S4: Schematic representation of RNAseq data mapped to the sequence of pSStarget-NT. A closeup view on the region encoding the sgRNA (nt: 6292-6429) shows reads mapped to the entire length of the sgRNA cassette, demonstrating expression of this locus.

Supporting Figure S5: Representative pictures of colony morphologies observed after plating transformation mix on CBA supplemented with 5% sheep blood and an increased Chloramphenicol concentration of 7.5μg/ml.

Supporting Table S1

Supporting Table S1: Oligonucleotides used in this study.
For the nucleotide sequences, uppercase letters denote the annealing portion of the primer, while lowercase letters indicate the non-annealing overhangs used for assembly of DNA fragments.

Short name	Name	Nucleotide sequence	Used for
P1	p15A-Tet Fw	agcgggacctactaGCGCTAGCGGAGTGTATACTG	pSStarget cloning
P2	p15A-Tet Rv	ctctcgagttgaTCAGGTCGAGGTGGCCCGGCT	pSStarget cloning
P3	ccdB_pSS2cloning_fw	taggttttattttatataattattgattgagaccCGCGTGGATCCGGCTTAC	pSStarget cloning
P4	ccdB_pSS2cloning_rv	acttgctatttctagctctaaaactgagaccCTGCAGACTGGCTGTGTATAAG	pSStarget cloning
P5	sg4_Fw	TGATGAGCGCGGCTACTGTTGTGAG	sgRNA cloning
P6	sg4_Rv	AAACCTCACAACAGTAGCCGCGCTC	sgRNA cloning
P7	sg6_Fw	TGATGAAGCTATAGAGTTTTCAACA	sgRNA cloning
P8	sg6_Rv	AAACTGTTGAAAACTCTATAGCTTC	sgRNA cloning
P9	sg42_Fw	TGATGTTTGATAGAAGGCTTGCGGA	sgRNA cloning
P10	sg42_Rv	AAACTCCGCAAGCCTTCTATCAAAC	sgRNA cloning
P11	Enolase_gRNA2_Fw	TGATGTACGACTACACTAAATTCGA	sgRNA cloning
P12	Enolase_gRNA2_Rv	AAACTCGAATTTAGTGTAGTCGTAC	sgRNA cloning
P13	Enolase_gRNA3_Fw	TGATGCGTCAAGAGCGATCATAGCG	sgRNA cloning
P14	Enolase_gRNA3_Rv	AAACCGCTATGATCGCTCTTGACGC	sgRNA cloning
P15	US_cps_Fw	ATCGTTGACTGTCCACCATTAG	RT construction
P16	US_cps_Rv	tctttttcatTTACTGTACTTGATTTTTCAATATCAACAAAGG	RT construction
P17	DS_cps_Fw	agtacagtaaATGAAAAAGATTCTATATCTCCATGCTGG	RT construction
P18	DS_cps_Rv	GGTGAGTTCGGAGTGACTAAG	RT construction
P19	US_sly_Fw	TATGTAAAGCACTAATAAACCGCTCTG	RT construction
P20	US_sly_Rv	gataaaactttTCTGGCAATGTATTATACTCTCTTAGC	RT construction
P21	DS_sly_Fw	cattgccagaAAGTTTTATCCTTTCATGTTTTCACTAATGAAATG	RT construction
P22	DS_sly_Rv	CAGATAGAGCTCAGTCCAGTG	RT construction
P23	US_lgt_Fw	CGTCTTCATCCATTCAAAC	RT construction
P24	US_lgt_Rv	gaggcttaatATGATTATTGAAATTTCTGTCTTG	RT construction
P25	DS_lgt_Fw	caataatcatATTAAGCCTCTTCTCTATTG	RT construction
P26	DS_lgt_Rv	CCCTAGCTTATGGTATAATATAATAAAAC	RT construction
P27	pUC57_Fw	TCAGAATCCGAGTGACAG	RT construction
P28	pUC57_Rv	GTGATTCCTTTACGGTGTC	RT construction
P29	EnolaseRT_US_Fw	ttggagggacaccgtaaaggaatcaTTAGAATACTCTAAATGATACATGC	RT construction
P30	EnolaseRT_US_Rv	CCAATCATCACTATCGAAG	RT construction
P31	EnolaseRT_DS_Fw	TTCACCAGCTTCGTAACC	RT construction
P32	EnolaseRT_DS_Rv	ttccaacctgtcactcggattctgaAACTTATTTCCGACGACTTC	RT construction
P33	pUC57_ins_Fw	GTTGAGTGTTGTTCCAGTTTGG	RT construction
P34	pUC57_ins_Rv	AGCGAGGAAGCGGAAGAG	RT construction
P35	eno_SDM_Fw	atctagggcaatcatggctcgatcGATAGCTTGTTGATCACG	RT construction
P36	eno_SDM_Re	gatcgagccatgattgccctagatGGTACTCCTAACAAAGGTAAATTG	RT construction
P37	eno_seq_Fw	CTTTAGCCATTTCGATAGC	RT construction
P38	eno_seq_Rv	GTAACAGCTGTTGGTGAC	RT construction

Supporting Table S2

Supporting Table S2: Overview of sequences and properties of all sgRNA spacers used in this study. The 20bp spacer sequence and the 3bp PAM directly adjacent to the target sequence are listed for each sgRNA, as well as the GC content and the on-target score calculated according to the method of Doench et al. (1)

sgRNA	Sequence	PAM	On-target score[1]	GC content (%)
sg4 (*sly*)	AGCGCGGCTACTGTTGTGAG	CGG	66.5	60
sg6 (*cps*)	GTTTATTAATGTGAAATACA	CGG	65.5	20
sg42 (*lgt*)	TTTGATAGAAGGCTTGCGGA	CGG	71.0	45
sg_*eno2*	TACGACTACACTAAATTCGA	AGG	6.6	35
sg_*eno3*	CGTCAAGAGCGATCATAGCG	CGG	83.3	55

Reference:

(1) Doench, J. G., Fusi, N., Sullender, M., Hegde, M., Vaimberg, E. W., Donovan, K. F., Smith, I., Tothova, Z., Wilen, C., Orchard, R., Virgin, H. W., Listgarten, J., & Root, D. E. (2016). Optimized sgRNA design to maximize activity and minimize off-target effects of CRISPR-Cas9. *Nature Biotechnology*, *34*(2), 184–191. https://doi.org/10.1038/nbt.3437

Supporting Figure S4

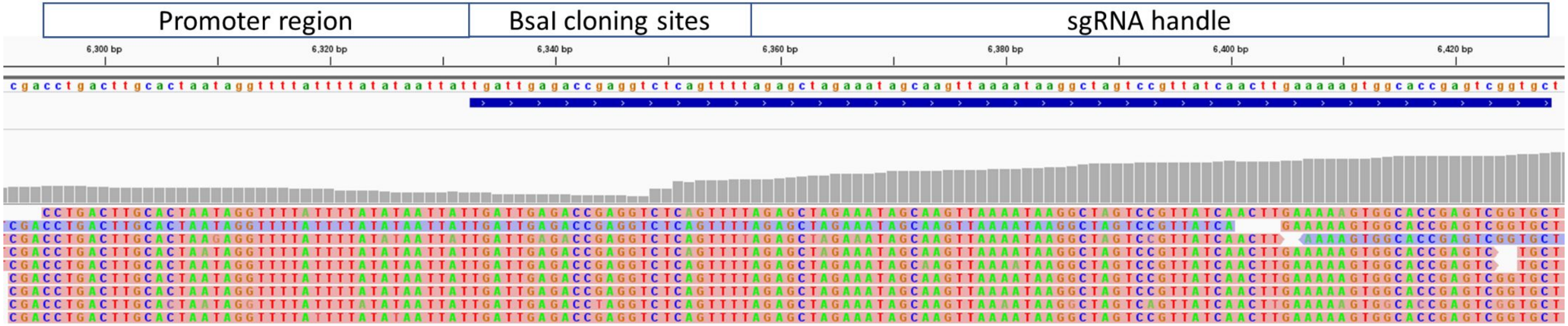

Supporting Figure S4: Schematic representation of RNAseq reads mapped to the sequence of pSStarget-NT. A closeup view on the region encoding the sgRNA (nt: 6292-6429) shows reads mapped to the entire length of the sgRNA cassette, demonstrating expression of this locus.

Supporting Figure S5

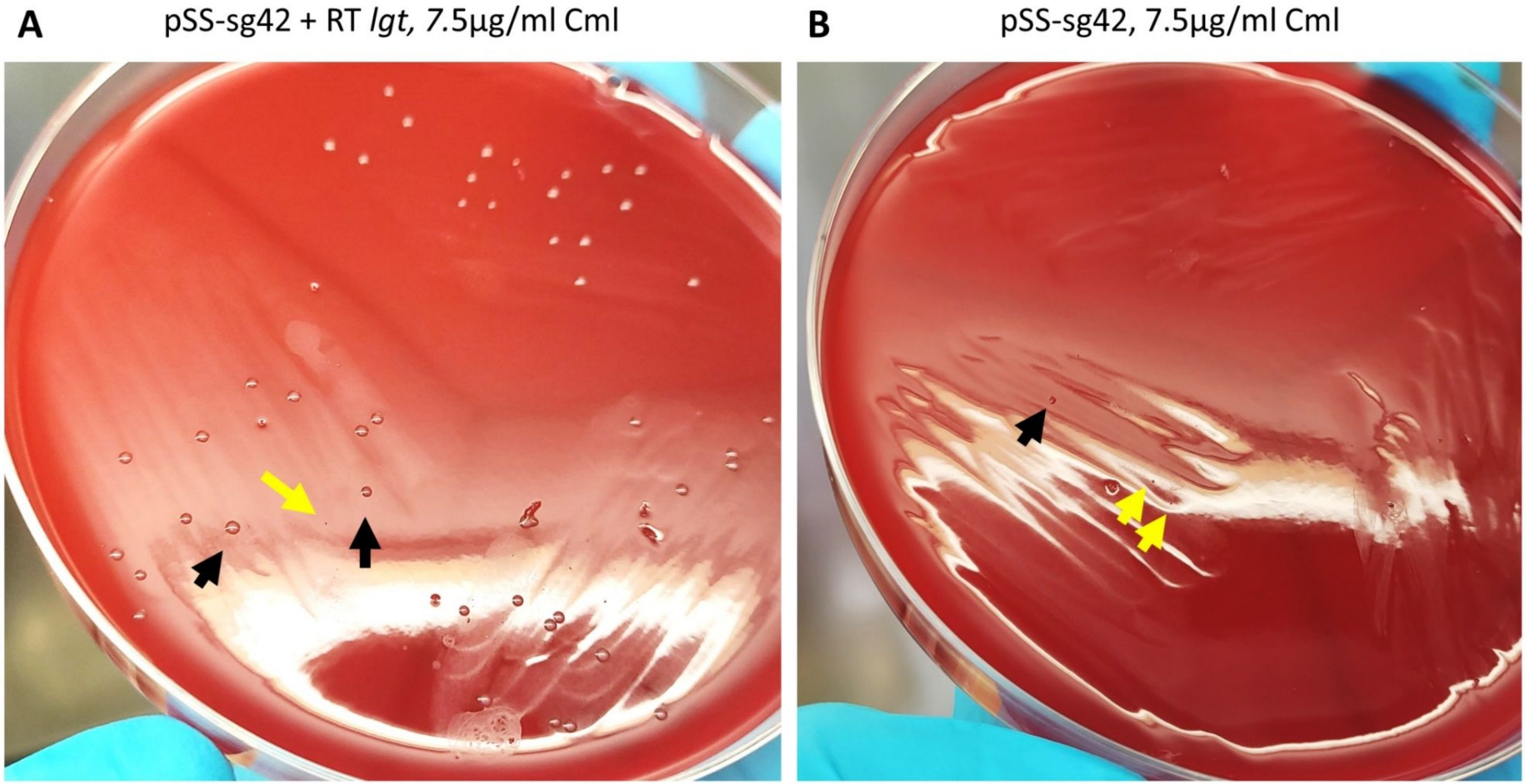

Supporting Figure S5: Representative pictures of colonies observed upon plating transformation mix on CBA supplemented with 5% sheep blood and an increased Chloramphenicol (Cml) concentration of 7.5µg/ml. The higher Cml concentration markedly reduced the number of small colonies formed (yellow arrows) but did not affect the number of normal sized colonies (black arrows). **(A)** *S. suis* P1/7 transformed with pSS-sg42 and the corresponding repair template (1.2µg each) forms very few SCVs when compared to the usual concentration of 5µg/ml. **(B)** *S. suis* P1/7 transformed only with pSS-sg42 (1.2µg) is used as a "background control" to assess the amount of SCVs formed in absence of a corresponding repair template. At the increased Cml concentrations the SCVs are evidently reduced in number as well as their size.
